# In the darkness of the polar night, scallops keep on a steady rhythm

**DOI:** 10.1038/srep32435

**Published:** 2016-08-31

**Authors:** Damien Tran, Mohamedou Sow, Lionel Camus, Pierre Ciret, Jorgen Berge, Jean-Charles Massabuau

**Affiliations:** 1 CNRS, UMR 5805 EPOC. Place du Dr. Peyneau, 33120, Arcachon, France; 2 Bordeaux University, UMR EPOC 5805 Place du Dr. Peyneau, 33120, Arcachon, France; 3 Akvaplan-niva, Fram center for Climate and the Environment, 9296 Tromso, Norway; 4 University Centre in Svalbard, Pb 156, N-9171 Longyearbyen, Norway; 5 UiT The Arctic University of Norway, Faculty of Biosciences, Fisheries and Economics, N-9037 Tromsø, Norway

## Abstract

Although the prevailing paradigm has held that the polar night is a period of biological quiescence, recent studies have detected noticeable activity levels in marine organisms. In this study, we investigated the circadian rhythm of the scallop *Chlamys islandica* by continuously recording the animal’s behaviour over 3 years in the Arctic (Svalbard). Our results showed that a circadian rhythm persists throughout the polar night and lasts for at least 4 months. Based on observations across three polar nights, we showed that the robustness and synchronicity of the rhythm depends on the angle of the sun below the horizon. The weakest rhythm occurred at the onset of the polar night during the nautical twilight. Surprisingly, the circadian behaviour began to recover during the darkest part of the polar night. Because active rhythms optimize the fitness of an organism, our study brings out that the scallops *C. islandica* remain active even during the polar night.

The Arctic region is undergoing major changes, including a rapid decline in ice cover and warming at a rate that is two to three times faster than that the rest of the planet^1^,^2^. These changes are likely to have numerous ecological consequences, such as shifts in distribution, behaviour and trophic interactions^3^,^4^,^5^,^6^. Until recently, the Arctic winter polar night was thought to be a “dead” period for life pending more favourable seasons. Therefore, an increase in the Artic temperature combined with a reduction in sea ice cover would deeply modify biological activity^7^. Previous theories on the polar night period (i.e., periods where the sun remains below the horizon for at least 24 h or more) described it as a period of reduced activity in the marine ecosystem^8^,^9^. In fact, the food web is described as a bottom-up process, in which the primary production level is reduced to near zero and inhibits the activity of the upper trophic levels. Recently, a new paradigm has challenged this classical theory. Several studies^10^,^11^,^12^,^13^ have revealed that the Arctic marine ecosystem can elicit high levels of activity and trophic interactions during the darkest period of the polar night, which is inconsistent with previous theories.

Circadian rhythms are a universal and fundamental characteristic of most phyla^14^. A molecular clock nested in each cell governs a wide range of 24-h rhythms from metabolism to behaviour^15^,^16^. The oscillation of the circadian clock is synchronized by ambient light-dark cycles. In temperate regions, this circadian rhythm is known to be adaptive and increases the fitness of the animal in its biotope^17^,^18^. However, when the environment is not characterized by a 24 h light/dark cycle, such as the environment of the Arctic region, it is uncertain whether an active circadian clock is beneficial to the organism. Closer to the poles, the seasonal changes in light intensity and duration become increasingly extreme throughout the year. Therefore, circadian rhythms may not be beneficial in polar regions^19^. For example, a previous study showed that the oyster *Crassostrea gigas* has a dual and weak endogenous circadian rhythm^20^. The activity of *C. gigas* is largely dependent on the cycle of the moon and the sun^21^, and in France, which is located at the latitude 45° N, oysters are nocturnal in the autumn and winter and diurnal in the spring and summer^20^.

In the present study, we continuously monitored the valve behaviour of the scallop in a high Arctic fjord, Kongsfjorden (Spitsbergen, Svalbard, 78° 56′ N), using an online and *in situ* system^20^,^21^. A total of 14 scallops (*Chlamys islandica)* were wired in parallel, and data were acquired 24 h, 7 days per week via the Internet. Valve activity is associated with gill filtration, which is necessary for respiration, nutrition and waste elimination. Therefore, monitoring valve gaping behaviour is a good proxy for measuring the global activity levels of bivalves in their ecosystem. This study was conducted over three years (1060 days) from 2012 to 2015. During this period, we primarily focused on the opening duration, amplitude and circadian rhythm of the valve. We recorded the valve activity during each season with a particular focus on three successive polar nights that lasted 116 days each. To improve our investigation of the valve activity and its relationship with the perception of light cues, the polar night period was divided into three well-defined and distinct sub-periods: civil, nautical and astronomical twilights. These sub-periods were based on the sun’s angle below the horizon and therefore consisted of different levels of low light and light intensity.

An aim of our study was to gain insight into polar night marine biology and more specifically into the function of the biological clock of a typical Arctic bivalve, the scallop *C. islandica* in the high Arctic. Our objective was to identify and characterize the circadian rhythm of a scallop from a high Arctic fjord (at latitude 79° N). We wanted to determine whether the scallop would continue to express a clear circadian rhythm (similar to bivalves in temperate zones) or express an ultradian rhythm. In addition, we wanted to determine if the circadian rhythm expressed during days with fluctuating light-dark periods would be maintained during the polar night and assess the role of the circadian rhythm in the function of the molecular clock and light perception. The results from this experiment will allow us to determine whether the polar night is a period of biological quiescence or a period of maintained activity for scallops.

## Results

### Circannual behaviour in Chlamys islandica

**Figure 1:**
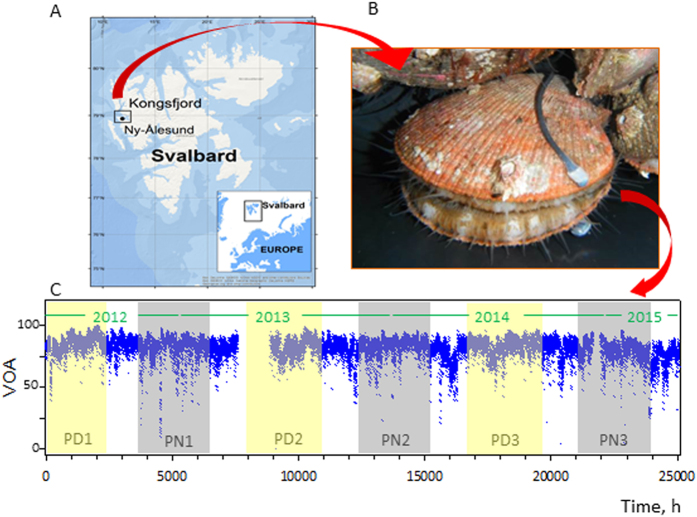
Locations of the scallops equipped with devices to record their valve behaviour. (**A**) Map showing the location of Ny-Alesund (78° 56′ N, 11°56′ E) where the study was conducted. The map was created using ArcGIS 10.2. The base map was built from a layer created by ‘Esri, DeLorme, GEBCO, NOAA NGDC, and other contributors’ available from https://www.arcgis.com/home/item.html?id=1e126e7520f9466c9ca28b8f28b5e500. (**B**) One of the 14 scallops (*C. islandica*) equipped with the electrodes of the HFNI valvometer biosensor. (**C**) Representative record of an individual scallop’s valve behaviour. In yellow: polar days (PD); in grey: polar nights (PN); in white: days with alternating light/dark periods (AL).

**Figure 2:**
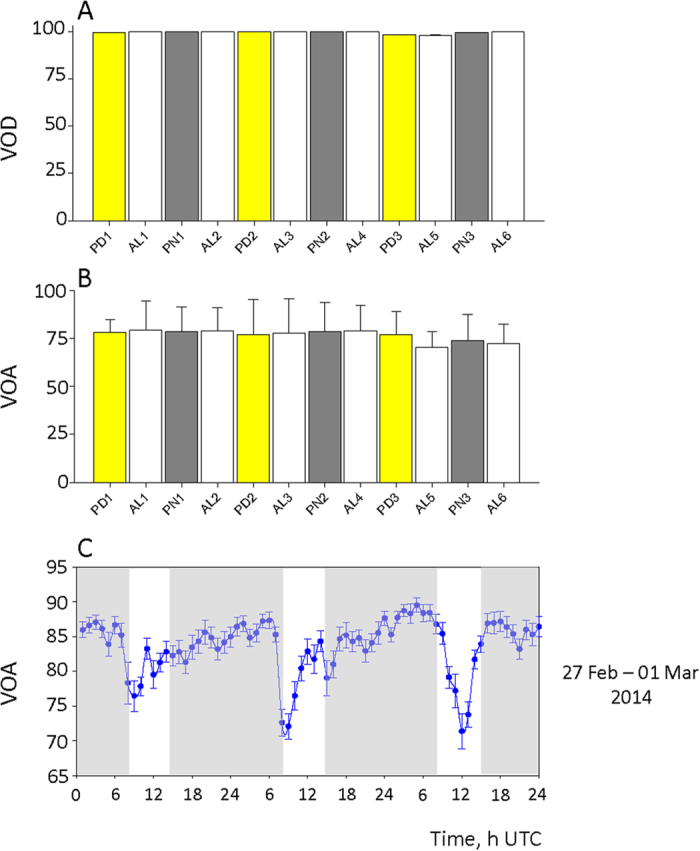
Group valve behavioural activity of *C. islandica.* (**A**) Mean (n = 14) valve opening duration (VOD, %) during the studied seasons. (**B**) Mean (n = 14) valve opening amplitude (VOA, %) during the studied seasons. For (**A**,**B**): yellow bars: polar days (PD); grey bars: polar nights (PN); white bars: days with alternating light/dark periods (AL). (**C**) Mean typical behaviour during the 3 days in the AL4 season (February 27^th^ -March 1^st^, 2014), which shows the VOA (%) ranging from 65% to 95%. Time zero: 13 h UTC. In grey: polar night periods.

Figure 1 shows a representative example of the 14 scallops that were studied in Svalbard (Fig. 1A) over1060 days (Fig. 1B, see timetable Fig. S11) and equipped with high-frequency non-invasive (HFNI) valvometer electrodes to record the valve activity (Fig. 1C). All of the studied scallops were similarly equipped (SI2). The valve opening amplitude (VOA) is shown as a function of time and season and was recorded for the polar days (PD), polar nights (PN) and alternating light/dark (AL) periods. A preliminary analysis showed that this bivalve species keeps its valve open continuously with low variation in the VOA (Fig. 2). The mean valve opening duration (VOD) was 99.4 ± 0.1% across all of the seasons studied in this experiment (Fig. 2A). No significant rhythm (*p* = 0.417) was detected as a function of the season. The VOA was measured for each season studied (Fig. 2B), and significant differences were not observed (*p* = 0.928) between the seasons, which had a group mean VOA of 76.7 ± 0.1%. These results show that the mean valve daily opening and duration does not change between the three seasons studied (AL, PN or PD), which suggests the absence of a circannual rhythm in the VOA. It is worthwhile noticing that no change of valve behaviour due to gametogenesis or spawning events was observed. Next, we focused on the daily valve activity level with regards to the mean VOA. Figure 2C shows that the variability in the VOA ranged between 65% and 95% during the 3-day focus period during the AL (Feb 27^th^ – March 1^st^, 2014; AL4). This pattern was representative of the typical diel VOA activity and the average variation in the VOA was 10–20%. Therefore, we conducted a chronobiological analysis of the diel VAO activity to study how the scallops organize their valve activity throughout the year.

### Seasonal circadian activity

**Figure 3:**
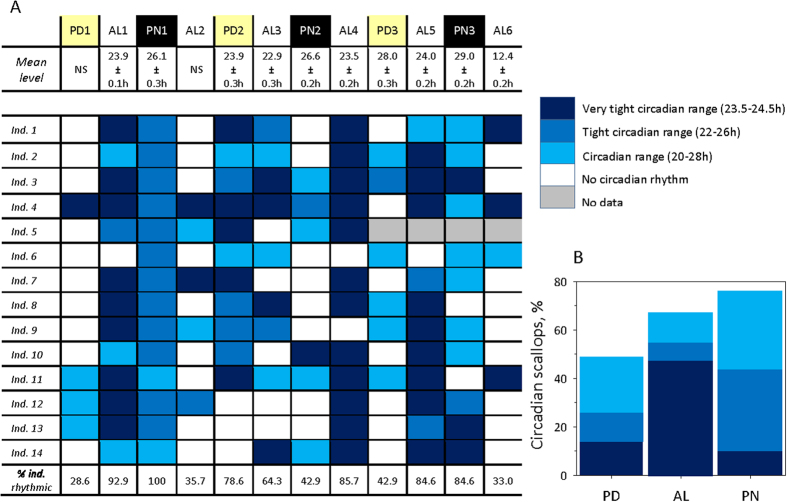
Circadian activity of *C. islandica.* (**A**) Diagram of the group and individual circadian valve behaviours. At the individual level, the percentage of rhythmic individuals in the circadian range (20–28 h) is shown for each studied season (3 PD, 3 PN and 6 AL). NS: indicates a non-significant period. For each scallop at each season, a colour tag indicates the circadian period range that is closest to 24 h (dark blue tag: 23.5–24.5 h), intermediate from 24 h (medium blue tag: 22–26 h), and farthest from 24 h (light blue tag: 20–28 h). The white tags indicate that no significant rhythm in the circadian range was observed. (**B**) Percentage of scallops with valve behaviour in the circadian range according to the different seasons. For the PD and PN periods, the mean represents an average for the 3 total seasons (1 per each year studied), whereas for the AL period, the mean represents an average for the 6 total seasons.

First, we investigated the occurrence of a circadian rhythm at both the group (n = 14 scallops) and individual level (Fig. 3). At the group level (Fig. 3A), the 6 AL periods generally demonstrated a rhythm close to 24 h, although AL2 did not have a circadian rhythm and AL6 displayed a tidal rhythm (12.4 h). In the PD periods studied at the group level, PD1 was not associated with any circadian rhythm; PD2 had a near 24 h rhythm; and PD3 had a 28 h rhythm. All of the PN periods displayed rhythmic activity, which was approximately 26 h for PN1 and PN2 and 29 h for PN3, which is similar to the circadian range. The details of the chronobiological analysis are shown in Fig. SI3.

At the individual level, significant variability in the percentage of rhythmic scallops was observed during all three periods. Figure 3B shows the individual variability during the different seasons. During the AL periods, 66.9% of the scallops were rhythmic, with 47.5% in the very tight circadian range (23.5–24.5 h). During the 3 PD periods, 49.7% of the scallops had a circadian rhythm, with 22.3% in the loose range (from 20–28 h). Surprisingly, during the 3 PN periods, 75.9% of the scallops were rhythmic and the majority of the scallops were in either the tight (22–26 h; 33.5%) or the loose circadian ranges (20–28 h; 32.3%). These data demonstrate that the rhythmic activity of the scallops during the polar night was similar to the activity during the AL periods; however, it was less synchronized to a tight circadian range.

### Circadian activity during the polar night

**Figure 4:**
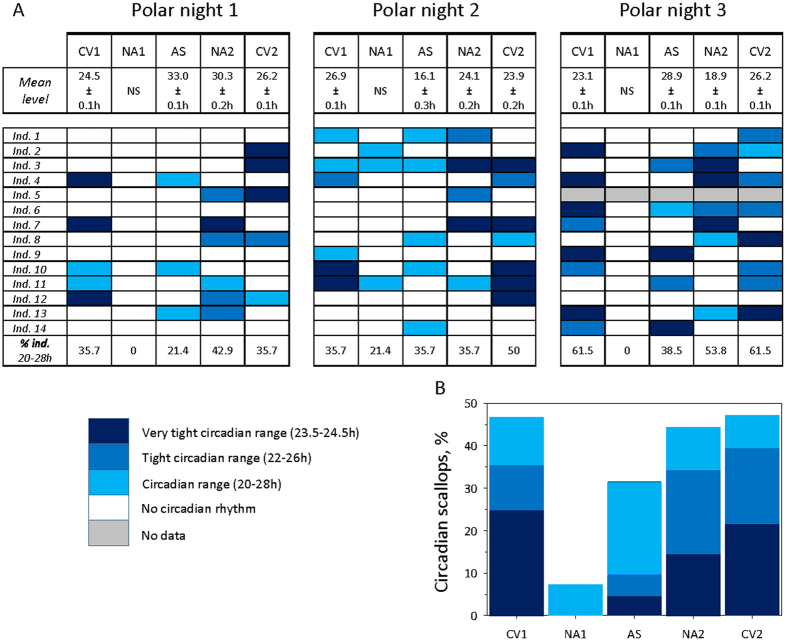
Focused analysis of the circadian activity of *C. islandica* during the polar night. (**A**) Diagrams of the group and individual circadian valve behavioural activity during the 3 polar nights studied (see S11 for a detailed timetable). Each polar night was sub-divided into 5 studied periods that corresponded to different twilight periods of increasing and decreasing levels of light irradiance: the civil twilight periods (CV1, CV2; the nautical twilight periods (NA1, NA2); and the astronomical twilight periods (AS) (see SI4). NS: indicates a non-significant period in the studied season. At the individual level, the percentage of rhythmic individuals in the circadian range (20–28 h) are shown for each twilight period (CV1, CV2, AS, NA1, NA2) and for each polar night studied. (**B**) Percentage of scallops with valve behaviour in the circadian range according to the different twilight periods. The mean percentage represents an average for the 3 polar nights studied.

As described earlier, the polar night at Ny-Alesund in Svalbard can be divided into different twilight periods. To refine our analysis on the behaviour of *C. islandica* during the polar night, we examined the strength of their circadian rhythm during the different twilight periods, which were defined according to the light irradiance. Five periods were investigated: 2 civil twilight periods, with decreasing (CV1) or increasing (CV2) light irradiance levels; 2 nautical twilight periods, with decreasing (NA1) or increasing (NA2) light irradiance levels; and 1 astronomical twilight (AS) periods, which represented the darkest period of the polar night. For details on the polar night timetable and the twilight periods, see Fig. SI4. Figure 4 shows the group and individual level data on the rhythmic behaviour of the scallops during the three polar nights covered in this study (from 2012–2015). At the group level, Fig. 4A shows that no significant rhythm was identified (Cosinor model, *p* > 0.05) during the nautical twilight, which begins the polar night. After the nautical twilight, the rhythm moved closer to 24 h as the level of light irradiance increases over time. During the three PN periods, the significant CV1 rhythms varied from 23.1 h to 26.9 h. In addition, significant rhythms were found (Cosinor model, *p* > 0.05) during the three PN for NA2, although with different periodicity. For PN2, the rhythm was close to 24 h, whereas during PN1 and PN3, the rhythm was equal to 30.3 h and 18.9 h, respectively, indicating that there was less synchronization between individuals as the light irradiance decreased. Finally, the lack of synchronization between individuals was reinforced during the AS periods. A significant rhythm (Cosinor model, *p* > 0.05) was detected at each AS period, although it was outside the circadian range at 33.0 h, 16.1 h and 28.9 h for PN1, PN2 and PN3, respectively. These findings highlight the loss of synchronization between the scallops during this darkest period of the polar night.

At the individual level (Fig. 4A), significant patterns were also identified. Notably, during several of the periods (civil, nautical and astronomical twilights), many of the scallops followed a rhythm close to 24 h. None of the scallops were rhythmic, even outside the circadian range, in NA1 during PN1 and PN3, and only 3 scallops were rhythmic during PN2, and they demonstrated a loose circadian rhythm (20–28 h). Figure 4B shows that 44.3 ± 8.6% and 49.1 ± 7.5% of the scallops were rhythmic during CV1 and CV2, respectively. During NA2 and AS, 44.1 ± 5.3% and 31.9 ± 5.2% of the scallops were rhythmic, respectively. In NA1, only 7.1 ± 7.1% of the scallops were rhythmic. Most notably, during the AS twilight, 31.9 ± 5.3% of the scallops presented a circadian rhythm. Furthermore, during the CV1 and CV2 civil twilights, the major circadian range was 23.5–24.5 h for 24.7 ± 7.2% and 21.8 ± 3.8% of the scallops, respectively. During NA2, 19.4 ± 4.6% of the scallops had a rhythm in the 22–26 h range. Finally, during the AS twilight, the major circadian rhythm was 20–28 h for 21.6 ± 8.1% of the scallops.

## Discussion

The aim of this research was to study the behaviour of a typical Arctic bivalve, the scallop *C. islandica* in the Svalbard and determine their circadian behaviour during the polar night. We recorded their valve activity in an ice-free Arctic fjord (Kongsfjorden, Spitsbergen, 78°56′ N) over three consecutive years. We found that the scallop’s valve remains open most of the time, with a high level of valve opening amplitude during equinox, but also during both the polar night and polar day. No circannual pattern in the VOA was observed. A daily VOA rhythm in the circadian range was present during the three polar nights studied. Moreover, the percentage of rhythmic scallops and the number of scallops that demonstrated rhythmicity close to an exact 24 h cycle were correlated to the different twilight periods of the polar night. The rhythm was more robust during the civil twilight period than the nautical and astronomical twilight periods. Interestingly, the period during which the bivalves were the least rhythmic was during the first nautical twilight period when light irradiance was decreasing (NA1) and not during the subsequent period (the astronomical twilight), which is the darkest period of the polar night in the Kongsfjorden.

This result shows that the scallops in the ice-free Kongsfjorden maintain a circadian rhythm for the majority of the year, which is similar behaviour to their counterpart species at lower latitudes in Norway (Tromso, 69°38′ N) and Russia (Murmansk, 68°57′ N) (personal data). This is also similar behaviour to their temperate zone counterpart species, such as the scallop *Chlamys varia* (personal data) and the oyster *Crassostrea gigas*^21^. The main exception occurred during a short period after the start of the polar night. These findings raise questions pertaining to the mechanism that drives activity during the polar night, the mechanism underlying the behaviour identified in this study, and the behaviour’s fitness benefits to the animal. These rhythms are typically invoked to increase the fitness of the animal in its environment. Maintaining a steady rhythm throughout the polar night suggests that the organism maintains an active metabolism and does not fall into a period of resting activity. Therefore, the present research provides significant insights into the active behaviour of this benthic Arctic species during the polar night, which extends the findings from recent reports^11^,^22^.

### Different behavioural strategies of animal activities in the Artic

Species may have different behavioural strategies depending on their specific metabolism and their geographical ecosystem. Certain species display 24 h behavioural activity with no overt rhythm, whereas others species express an apparent and clear rhythm^19^. In polar conditions, questions arise as to why a species would maintain a circadian rhythm in an environment that does not always fluctuate on a 24-h light-dark cycle. Specifically, how would an active circadian clock be beneficial to the species? A “true” rhythm differs from masking effects, which are a direct response to environmental cues^23^, and is created by a molecular clock that is included in all cells of the organism and entrained and synchronized by external cues called zeitgebers^24^,^25^. If the clock is robust, this rhythm persists in constant conditions without the need for entrainment and synchronization. The circadian rhythm observed in *C. islandica* during the polar night suggests that (i) either the scallop is equipped with a robust clock that allows it to maintain a rhythm in the circadian range without being synchronized by light, (ii) the scallop is able to maintain synchronization using low light irradiance cues, or (iii) the circadian clock of the scallop is not active during the polar night but the scallop directly responds to low intensity light cues. Moreover, the regular daily fluctuations in light intensity (even small increases and decreases in amplitude) appear to be important for maintaining the scallop’s rhythm. In fact, the number of rhythmic individuals was higher during NA2 (44.1%) when the light irradiance increases compared with that during NA1 (7.1%) when the light irradiance decreases. At the start of the astronomical twilight (the period in our study when the polar night is the darkest), the percentage of rhythmic scallops again increased to 31.9% of individuals. These results suggest that during the darkest part of the polar night, the rhythmic activity resumes to optimize the organism for when the light reappears. Therefore, the nautical twilight (NA1), the period just before the astronomical twilight, could be the period when the bivalves are the least active and could correspond to a physiological resting time. A question remains, which is the existence of other zeitgeber besides the sun light to explain the circadian synchronization although there is no obvious candidate. The tidal periodicity of 12.4 h is evidently inappropriate whereas the lunar-day cycle of 24.8 h, driven by the moonlight, is not a good candidate because the circadian rhythm remains even in new moon. Moreover, during the NA1 period, the lunar-day cycle exists and no 24.8 h rhythm appears in scallops. A food availability cycle is another putative circadian zeitgeber. The scallops are filter-feeders, usually they eat unicellular phytoplankton. Studies in Arctic and especially in Svalbard^11^,^12^ have shown that the photosynthetic activity of phytoplankton falls to zero during the darkest period of the polar night. At the end of polar night the alternative food sources are essentially the heterotrophic microbial plankton, which is not a good candidate as putative circadian zeitgeber. Although there is a lack of studies on biological rhythms during the polar night, we suggest different putative mechanisms may underlie this behaviour in specific ice-free regions of high Arctic, especially during the dark period (see Fig. SI5).

### Light perception and consequences

The studied scallops were grown and maintained in shallow water (4-5 m depth), even during the polar night^11^; together with the present behavioural data, this suggests that the environment is adequate for the physiological status and welfare of the species. However, it is unclear whether the ambient light during the polar night at greater ocean depths is a sufficient zeitgeber to entrain the circadian clock or an adequate visual cue to synchronize the behaviour in *Chlamys islandica*. Studies of diel vertical migration (DVM) of zooplankton in Kongsfjorden^10^,^13^ have shown that the krill *Meganyctiphanes norvegica* and the copepod *Calanus ssp*. have sufficient light at 60 m to maintain light-mediated behaviour. Scallops have complex eyes relative to other bivalves. Similar to other *Chlamys* species, *Chlamys islandica* are equipped with eyes distributed around the margin of the middle fold of the mantle. Although their eyes are more primitive than that of mammals, they contain a cornea, lens, mirror and retina that are sensitive to light variations^26^,^27^,^28^. In addition, to increase light perception, *C. islandica* may have developed a visual range more adapted to polar conditions to detect low intensities of light irradiance during the twilight periods and allow the bivalve to synchronize for longer periods of time. Similar effects have been reported for Arctic reindeer (*R. tarandus),* whose visual range extends into the ultraviolet (UV) range^29^, which is dissimilar to the more southern varieties of the species. Polar regions have a proportionally higher level of UV light because of the scatter and reflection of light from the ice cover and the low position of the sun on the horizon. Diffuse radiation, which is in the UV range and outside of the human visible spectrum, is the dominant component of the Arctic irradiance and contributes to the blue coloration of the Arctic twilight observed during mid-day in mid-winter and during dawn and dusk in spring and autumn^30^. Water reflects approximately 5% of the UV light, whereas snow and ice cover reflects approximately 85%. In terms of global warming changes, we hypothesize propose that a decrease in ice thickness could modify the light environment of marine organisms, even during the polar night. An impact of changes in the sea ice cover has already been reported^31^ for the Arctic cockle (*Clinocardium ciliatum*); the growth rate of the Artic cockle was dependent on sea ice cover and reduced when ice covered the water. Conversely, it was observed that the growth rate for *C. islandica* can be constant throughout the year in the ice-free Kongsfjorden^11^. Therefore, we suggest the hypothesis that the circadian activity of the scallop was different 10–15 years ago when ice was present during winter in Kongsfjorden. As such, the present findings could be the result of a lack of ice cover and may represent a transient snapshot of the scallop’s behaviour caused by the deep and rapid modifications of the Artic region because of global warming. An unexpected finding could be that climate change in the polar ecosystem could increase the strength of the rhythmic activity of marine inhabitants and limit the period of resting time during the polar night. We can hypothesize that a decrease in ice cover during the polar night could act as a shift of latitude in terms of circadian activity described for other more temperate species^18^. In fact, an increase in the robustness of the apparent circadian behaviour was observed in the reindeer *R. tarandus*^32^ and the bird *L. mutus hyperboreus*^33^ with a decrease in latitude from 78° N to 70° N because of changes in the light regime of the new climate.

### Life in the polar night

The classical view of Arctic marine ecology^8^,^9^ assumed that during the polar night, biological processes were strongly reduced throughout the ecosystem because of low food availability and the decrease in light irradiance. For example, strategies to survive the long period of the polar night include dormant or diapause stages, which involve a state of reduced metabolism combined with the use of lipid energy reserves^34^,^35^. However, recent studies have challenged this classical paradigm^12^, especially in the pelagic food webs. The photosynthetic activity of phytoplankton is restricted to a low level because of the lack of light; therefore, phytoplankton cannot provide the first level of the food chain. However, the microbial plankton communities, which are involved in the flux of nutrients and carbon into the marine ecosystem, are still able to consume biogenic carbon produced during the previous light season^36^. Therefore, it would appear that heterotrophic activity could compensate for photosynthetic activity, even though the quality is lower in terms of food supply, to ensure the primary level of an active food web^37^,^38^,^39^. Recent studies have also shown a high level of activity during the polar night in terms of the DVM of zooplankton, amphipods, krill and jellyfish^10^,^40^ as well as other active swimmers, such as shrimp, fish and squid^40^. Finally, visual predators, such as seabirds, remain active and dive to catch fish during most of the polar night^41^. Benthic species have been poorly studied during the polar night, and little information is available on the strategies adopted by these species during winter. Although studies in Greenland have shown a reduction in shell growth in Arctic bivalves during the polar night^31^,^42^, these finding are modulated by recent studies in Svalbard showing a possible absence of reduced growth in the scallop *C. islandica*^11^. Continuous growth throughout the year was also found in the Artic amphipod *Onisimus litoralis*^43^.

## Conclusions

For the first time, a high-frequency recording system was deployed *in situ* to measure the valve activity of an Arctic bivalve, the scallop *C. islandica*, over a continuous period of approximately 3 years. This *in situ* data were used to investigate their behaviour and to determine their biological rhythm during the specific light periods that occur in a recently ice-free fjord in Svalbard. We discovered that the valve behaviour of *C. islandica* follows a circadian pattern in the majority of the selected individuals. This pattern is maintained even during the polar night, suggesting a sustained level of activity of *C. islandica*. This supports the notion that the polar night does not represent an inevitably quiescent period during but could be rather a moment of intense activity for these scallops. Similar studies should be conducted under ice-covered Arctic conditions (such as those observed at different longitudes) with different UV light penetration to determine whether the expression of biological rhythms is different from what was observed in our study. The results of these studies could shed new light on the possible impact of global warming, which may indicate that changes in the Artic climate could at least initially enhance biological rhythms for the scallops’ that inhabit the Arctic ecosystem.

## Material and Methods

### Study area and general conditions

This study was conducted using 14 scallops (*Chlamys islandica*). All of the specimens were collected during October 2011 in a scallop bed located south of Moffen Island and north of Svalbard using a dredge deployed from the research vessel RV Helmer Hanssen. Then, the scallops were acclimated during 6 months near the experimental site before the valve behaviour monitoring. The specimens were 53.6 ± 1.2 mm in length, 48.9 ± 1.1 mm in width and 15.4 ± 0.5 mm in thickness. The scallops were placed in individual boxes in a ballasted cage (50 × 100 cm), which was then lowered to a depth of 3 m at low tide to the sea floor under the old pier in Ny-Alesund (78° 56′ N, 11°56′ E), Kongsfjorden, Spitsbergen Island, Svalbard (Fig. 1A).

### Timetable and description of the seasons analysed for valve behavioural activity

This study was performed from 05/21/2012 to 04/16/2015, which corresponded to 1060 days of measurements. The analyses included 3 periods of midnight sun (“polar day”; PD1, PD2, and PD3), 3 periods of polar night (PN1, PN2, and PN3) and 6 periods of autumn/spring in which the light regime alternates between light and dark (AL1 to AL6; see timetable S11). The analyses were focused on the 3 polar nights. In the present experiment, the polar nights were separated into five successive periods (CV1, NA1, AS, NA2 and CV2) according to the evolution of light irradiance (see SI4). These periods corresponded to the two civil twilight periods (CV1 and CV2), which corresponded to the days of the solar culmination during the civil twilight; the nautical twilight periods (NA1, NA2), which corresponded to the days of the solar culmination occurring during the nautical twilight; and the astronomical twilight period (AS), which corresponded to the days of the solar culmination occurring during the astronomical twilight.

### Circadian ranges

To test the strength of the rhythm and its synchronization at low levels of light, three different ranges of circadian periods were used: the classical loose circadian range (20–28 h; light blue tag) and circadian ranges of 22–26 h and 23.5–24.5 h (medium and dark blue tags, respectively), which are more tightly centred on an exact 24 h cycle.

### Chlamys islandica behavioural measurements

Valve movement behaviour was monitored *in situ* with a HFNI valvometer biosensor^21^. Briefly, lightweight electromagnets (0.1 g) were glued on both valves of each individual animal (Fig. 1B), and flexible wires linked the electrodes to the biosensor. This technology is based on the measurement of voltage variations produced via the electromagnetic field between the two electromagnets. The sampling frequency for each individual was 0.62 Hz. The signal was recorded using a custom acquisition card, and the data were automatically transmitted daily to a data processing centre in the Arcachon Marine Biological Station (France) using cellular and Internet networks. The data were reported daily on the Molluscan Eye website (http://molluscan-eye.epoc.u-bordeaux1.fr/).

The valve behaviour endpoints included the valve opening duration (VOD) and the valve opening amplitude (VOA) of each individual and the group. A VOD equal to 100% indicated that the scallop’s valve was open for the entire time studied, and a VOA equal to 100% indicated that the scallop’s valve was open at its maximum gaping amplitude for the entire time studied, whereas a VOA equal to 0% indicated that the scallop’s valve was closed.

### Data treatment and statistical analysis of the rhythmic activity

The mean VOA was calculated to characterize the rhythmic activity. Double-plotted actograms (each line represents 2 days) were produced using Chronos-Fit 1.06^44^. Activity levels above the average for the day are represented by a black section, whereas values below the 24 h average are represented by a white section.

Chronobiological analyses were performed using the Time Series Analysis Serial Cosinor 6.3 software (http://www.euroestech.net/mainuk.php). Different steps were performed to validate a rhythm in the scallops^20^,^45^,^46^. Briefly, the quality of the data set was assessed by controlling for the absence of randomness using the autocorrelation diagram and the absence of a stationary character using a partial autocorrelation function (PACF) calculation^47^. The periodicities in the recorded data were tested using the spectral method of the Lomb and Scargle periodogram^48^. These methods provide a threshold of probability (*p*-value = 0.95) that defines the limit below which the signal can be regarded as “noise.” The confidence interval of the period was determined using methods described by Halberg^49^. When a period was statistically validated, the rhythmicity was modelled using the Cosinor model, which uses a cosine function calculated by a regression^50^,^51^. The model for a given period is written as: Y(t) = A.cos(2πt/τ + φ) + M + ϵ(t), where A is the amplitude, φ is the acrophase, τ is the period, M is the mesor, and ϵ is the relative error^45^. To validate the model, two tests were performed: the elliptic test^51^, must which must be rejected, and the probabilities (*p*-value) for the null amplitude hypothesis, which must be lower than 0.05. For a set of data, several significant periodicities could occur. To identify secondary periodicities, we reinjected the previously calculated residues of the Cosinor model and then repeated the entire procedure. This procedure was repeated for a maximum of 10 times.

In this experiment, the validation of a significant rhythm was complete when the entire procedure, i.e., the quality checks of the data, the identification of a significant period by spectral analysis and the statistical validation of the Cosinor model, was performed.

In the present study, we focused on the circadian periods ranging from 20 h to 28 h.

The results are presented as the mean ± SE. For all of the statistical tests, a *p*-value = 0.05 was considered statistically significant.

## Additional Information

**How to cite this article**: Tran, D. *et al*. In the darkness of the polar night, scallops keep on a steady rhythm. *Sci. Rep.* **6**, 32435; doi: 10.1038/srep32435 (2016).

## Supplementary Material

Supplementary Information

## References

[b1] Meier W. N. . Arctic sea ice in transformation: a review of recent observed changes and impacts on biology and human activity. Rev. Geophys. 51, 185–217 (2014).

[b2] Grambling C. Arctic impact. Is the melting Arctic really bringing frigid winters to North America and Eurasia? Science 347, 818–821 (2015). 25700497 10.1126/science.347.6224.818

[b3] Wassmann P., Duarte C. M., Agusti S. & Sejr M. Footprints of climate change in the Arctic marine ecosystem.Glob. Change Biol. 17, 1235–1249 (2011).

[b4] Wassmann P. . The contiguous domains of Arctic Ocean advection: trails of life and death. Prog. Oceanog 139, 42–65 (2015).

[b5] Bradshaw W. E. & Holzapfel C. M. Light, time, and the physiology of biotic response to rapid climate change in animals. Annu. Rev. Physiol. 72, 147–166 (2010). 20148671 10.1146/annurev-physiol-021909-135837

[b6] Saikkonen K. . Climate change-driven species’ range shifts filtered by photoperiodism. Nat. Clim. Chang 2, 234–242 (2012).

[b7] Kortsch S. . Climate-driven regime shifts in Arctic marine benthos. Proc. Natl. Acad. Sci. USA 109, 14052–14057 (2012). 22891319 10.1073/pnas.1207509109 PMC3435174

[b8] Piepenburg D. Recent research on Arctic benthos: common notions need to be revised. Polar Biol. 28, 733–755 (2005).

[b9] Smetacek V. & Nicol S. Polar ocean ecosystems in a changing world. Nature 437, 362–368 (2005). 16163347 10.1038/nature04161

[b10] Berge J. . Diel vertical migration of Arctic zooplankton during the polar night. Biol. Lett. 5, 69–72 (2009). 18948249 10.1098/rsbl.2008.0484 PMC2657746

[b11] Berge J. . Unexpected levels of biological activity during the polar night offer new perspectives on a warming Arctic. Curr. Biol. 25, 2555–2561 (2015). 26412132 10.1016/j.cub.2015.08.024

[b12] Berge J. . In the dark: A review of ecosystem processes during polar night. Prog. Oceanogr. 139, 258–271 (2015).

[b13] Cohen J. H. . Is ambient light during the high Arctic polar night sufficient to act as a visual cue for zooplankton? Plos One 10(6), e0126247. doi:10.1371/journal.pone.0126247 (2015). 26039111 PMC4454649

[b14] Bell-Pedersen D. . Circadian rhythms from multiple oscillators: lessons from diverse organisms. Nat. Rev. Genet 6, 544–556 (2005). 15951747 10.1038/nrg1633 PMC2735866

[b15] Zhang E. E. & Kay S. A. Clocks not winding down: Unravelling circadian networks. Nat. Rev. Mol. Cell. Biol. 11, 764–776 (2010). 20966970 10.1038/nrm2995

[b16] Green C. B., Takahashi J. S. & Bass J. The meter of metabolism. Cell 134, 728–742 (2008). 18775307 10.1016/j.cell.2008.08.022 PMC3760165

[b17] Yerushalmi S. & Green R. M. Evidence for the adaptive significance of circadian rhythms.Ecol. Lett. 12, 970–981 (2009). 19566794 10.1111/j.1461-0248.2009.01343.x

[b18] Hut R. A., Paolucci S., Dor R., Kyriacou C. P. & Daan S. Latitudinal clines: an evolutionary view on biological rhythms. Proc. R. Soc. B. 280, 20130433, doi:10.1098/rspb.2013.0433 (2013). PMC3712436 23825204

[b19] Bloch G., Barnes B. M., Gerkema M. P. & Helm B. Animal activity around the clock with no overt circadian rhythms: patterns, mechanisms and adaptive value. Proc. R. Soc. B. 280, 20130019, doi:10.1098/rspb.2013.0019 (2013). PMC3712434 23825202

[b20] Tran D. . Field chronobiology of a molluscan bivalve: How the moon and sun cycles interact to drive oyster activity rhythms. Chronobiol. Int. 28, 307–317 (2011). 21539422 10.3109/07420528.2011.565897

[b21] Mat A. M., Massabuau J. C., Ciret P. & Tran D. Evidence for a plastic dual circadian rhythm in the oyster *Crassostrea gigas*. Chronobiol. Int. 29, 857–867 (2011). 22823869 10.3109/07420528.2012.699126

[b22] Last K. S., Hobbs L., Berge J., Brierley A. S. & Cottier F. Moonlight Drives Ocean-Scale Mass Vertical Migration of Zooplankton during the Arctic Winter. Curr.Biol. 26, 244–251 (2016). 26774785 10.1016/j.cub.2015.11.038

[b23] Mrosovsky N. Masking: history, definitions, and measurement. Chronobiol. Int. 16, 415–429 (1999). 10442236 10.3109/07420529908998717

[b24] Roenneberg T. & Merrow M. The network of time: Understanding the molecular circadian system. Curr. biol. 13, 198–207 (2003). 10.1016/s0960-9822(03)00124-6 12620213

[b25] Young M. W. & Kay S. A. Time zones: a comparative genetics of circadian clocks. Nat. Rev. Genet. 2, 702–715 (2001). 11533719 10.1038/35088576

[b26] Dakin W. J. The eye of Pecten. Q. J. Microsc. Sci. 55, 49–112 (1910).

[b27] Butcher E. O. The formation, regeneration and transplantation of eyes in Pecten (*Gibbus borealis*). Biol. Bull. 59, 154–164 (1930).

[b28] Morton B. The function of the pallial eyes withing the Pectinidae, with a description of those present in *Patinapecten yessoensis*. In The Evolutionary Biology of the Bivalvia (ed. Harper E. M., Taylor J. D. & Crane J. A.), 247–255. Geological Society of London Special Publications 177 (2000).

[b29] Hogg C. . Arctic reindeer extend their visual range into the ultraviolet. Exp. Biol. 214, 2014–2019 (2011). 10.1242/jeb.053553 21613517

[b30] Weatherhead B., Tanskanen A. & Stevermer A. Ozone and Ultraviolet Radiation. In Arctic Climate Impact Assessment report (ed. Walch J. E.). Cambridge University Press, New York, 151–182 (2005).

[b31] Sejr M. K., Blicher M. E. & Rysgaard S. Sea ice cover affects inter-annual and geographic variation in growth of the Arctic cockle *Clinocardium ciliatum* (Bivalvia) in Greenland. Mar. Ecol. Prog. Ser. 389, 149–158 (2009).

[b32] van Oort B. E. H., Tyler N. J. C., Gerkema M. P., Folkow L. & Stokkan K.-A. Where clocks are redundant: weak circadian mechanisms in reindeer living under polar photic conditions. Naturwissenschaften 94, 183–194 (2007). 17131139 10.1007/s00114-006-0174-2

[b33] Reierth E., Vant Hof T. J. & Stokkan K.-A. Seasonal and daily variation in plasma melatonin in the high-arctic Svalbard Ptarmigan (*Lagopus Mutus Hyperboreus*). J. Biol. Rhythms. 14, 314–319 (1999). 10447312 10.1177/074873099129000731

[b34] Varpe O., Jorgensen C., Tarling G. A. & Fiksen O. The adaptive value of energy storage and capital breeding in seasonal environments. Oikos 118, 363–370 (2009).

[b35] Varpe O. Fitness and phenology: annual routines and zooplankton adaptations to seasonal cycles. J. Plankton Res. 34, 267–276 (2012).

[b36] Calbet A. & Landry M. R. Phytoplankton growth, microzooplankton grazing, and carbon cycling in marine systems. Limnol. Oceanogr. 49, 51–57 (2004).

[b37] Seuthe L., Rokkan Iversen K. & Narcy F. Seasonal microbial processes in a high-latitude fjord (Kongsfjorden, Svalbard): II. Ciliates and dinoflagellates. Polar Biol. 34, 751–766 (2011).

[b38] Rokkan Iversen K. & Seuthe L. Seasonal microbial processes in a high-latitude fjord (Kongsfjorden, Svalbard): I. Heterotrophic bacteria, picoplankton and nanoflagellates. Polar Biol. 34, 731–749 (2011).

[b39] Leu E. . Arctic spring awakening – steering principles behind the phenology of vernal ice algal blooms. Prog. Oceanogr. 139, 151–170 (2015).

[b40] Webster C. . Moonlit swimming: vertical distributions of macrozooplankton and nekton during the polar night. Polar Biol. 38, 1–11 (2013).

[b41] Gremillet D. . Cormorants dive through the Polar night. Biol. Lett. 1, 469–471 (2005). 17148235 10.1098/rsbl.2005.0356 PMC1626366

[b42] Ambrose W. G. . Growth line deposition and variability in growth of two circumpolar bivalves (*Serripes groenlandicus*, and *Clinocardium ciliatum*). Polar Biol. 35, 345–354 (2012).

[b43] Nygård H., Berge J., Soreide J. E., Vihtakari M. & Falk-Petersen S. The amphipod scavenging guild in two Arctic fjords: seasonal variations, abundance and trophic interactions. Aquat. Biol. 14, 247–264 (2012).

[b44] Zuther P., Gorbey S. & Lemmer B. Chronos-Fit 1.06, http://www.ma.uniheidel (2009).

[b45] Gouthière L., Claustrat B., Brun J. & Mauvieux B. Complementary methodological steps in the analysis of rhythms: search of periods, modelling. Examples of plasma melatonin and temperature curves. Pathol. Biol. 5, 285–289 (2005). 15939140 10.1016/j.patbio.2004.12.025

[b46] Gouthière L., Mauvieux B., Davenne D. & Waterhouse J. Complementary methodology in the analysis of rhythmic data, using examples from a complex situation, the rhythmicity of temperature in night shift workers. Biol. Rhythm. Res. 36, 177–193 (2005).

[b47] Box G. E. P., Jenkins G. M. & Reinsel G. C. Time Series Analysis: Forecasting and Control Third edition. New York, Prentice Hall, (1994).

[b48] Scargle J. D. Studies in astronomical time series analysis 2. Statistical aspects of spectral-analysis of unevenly spaced data. Astrophys. J. 263, 835–853 (1982).

[b49] Halberg F. Chronobiology. Ann. Rev. Physiol. 31, 675–726 (1969). 4885778 10.1146/annurev.ph.31.030169.003331

[b50] Nelson W., Tong Y. L., Lee J. K. & Halberg F. Methods for Cosinor-rhythmometry. Chronobiologia 6, 305–323 (1979). 548245

[b51] Bingham C., Arbogast B., Guillaume G. C., Lee J. K. & Halberg F. Inferential statistical methods for estimating and comparing cosinor parameters. Chronobiologia 9, 397–439 (1982). 7168995

