# Supplementary material for: In the darkness of the polar night, scallops keep on a steady rhythm
Source: Sci Rep. 2016 Aug 31;6:32435. doi: 10.1038/srep32435 (PMC5006026; doi:10.1038/srep32435)
Supplement: Supplementary Information [file srep32435-s1.pdf]

Supplement information

In the darkness of the polar night, scallops keep on a steady rhythm

Damien Tran[1,2*], Mohamedou Sow[2], Lionel Camus[3,5], Pierre Ciret[1,2], Jorgen Berge[4,5], and Jean-Charles Massabuau[1,2]

[1]CNRS, UMR 5805 EPOC. Place du Dr. Peyneau, 33120, Arcachon, France.

[2]Bordeaux University, UMR EPOC 5805 Place du Dr. Peyneau, 33120, Arcachon, France.

[3]Akvaplan-niva, Fram center for Climate and the Environment, 9296 Tromso, Norway.

[4]UiT The Arctic University of Norway, Faculty of Biosciences, Fisheries and Economics, N-9037 Tromsø, Norway.

[5] University Centre in Svalbard, Pb 156, N-9171 Longyearbyen, Norway.

* to whom correspondence should be addressed. UMR 5805 EPOC, Place du Dr Peyneau, 33120, Arcachon, France.

E.mail: d.tran@epoc.u-bordeaux1.fr

Fax : +33 56549383 Tel : +33 562239237

Supplemental figures and tables

SI1. *Timetable of the study*.

The dates of the different seasons studied, using the website www.timeanddate.com/sun/norway/ny-alesund, during the experiment lasting 1060 days, from 21 May 2012 to 16 April 2015. In total, 12 seasons studied: 3 polar days (PD1, PD2, PD3); 3 Polar nights (PN1, PN2, PN3) and 6 seasons of light/dark alternations around the equinoxes (AL1 to AL6). During these 1060 days of monitoring, 68 days of record are missing due to problem of data transfer.

Seasons	**Polar day 1**	**LD Alterna -tion 1**	**Polar night 1**	**LD Alterna -tion 2**	**Polar day 2**	**LD Alterna -tion 3**	**Polar night 2**	**LD Alterna -tion 4**	**Polar day 3**	**LD Alterna -tion 5**	**Polar night 3**	**LD Alterna -tion 6**
Abbreviation	PD 1	AL1	PN1	AL2	PD 2	AL 3	PN 2	AL 4	PD 3	AL 5	PN3	AL6
Dates	21/05/12<br>26/08/12<br>*97 days*	27/08/12<br>23/10/12<br>*55 days*	24/10/12<br>16/02/13<br>*116 days*	17/02/13<br>15/04/13<br>*58 days*	16/04/13<br>26/08/13<br>*133 days*	27/08/13<br>24/10/13<br>*56 days*	25/10/13<br>17/02/14<br>*116 days*	18/02/14<br>16/04/14<br>*58 days*	17/04/14<br>26/08/14<br>*132 days*	27/08/14<br>24/10/14<br>*57 days*	25/10/14<br>17/02/15<br>*116 days*	18/02/15<br>16/04/15<br>*58 days*
Missing data	_	_	_	8 days<br>08/04 -<br>15/04/13	47 days<br>16/04 –<br>02/06/13	_	_	_	_	_	13 days<br>13/11 –<br>25/11/14	_

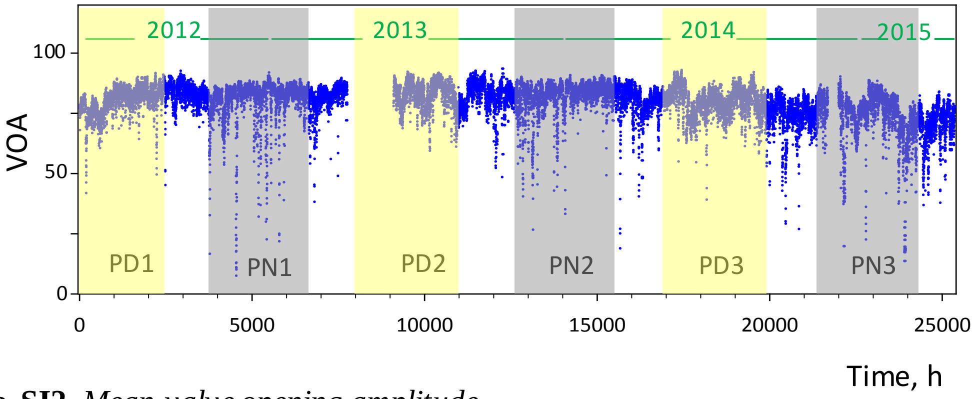

Figure SI2. *Mean valve opening amplitude.*

Mean record of valve behavior of the 14 *C. islandica* studied. VAO (percentage of valve opening amplitude) measured during the whole experiment lasting 1060 days, from 21 May 2012 to 16 April 2015. In yellow: polar days (PD); in grey: polar nights (PN); in white: days with alternation of light/dark conditions (AL).

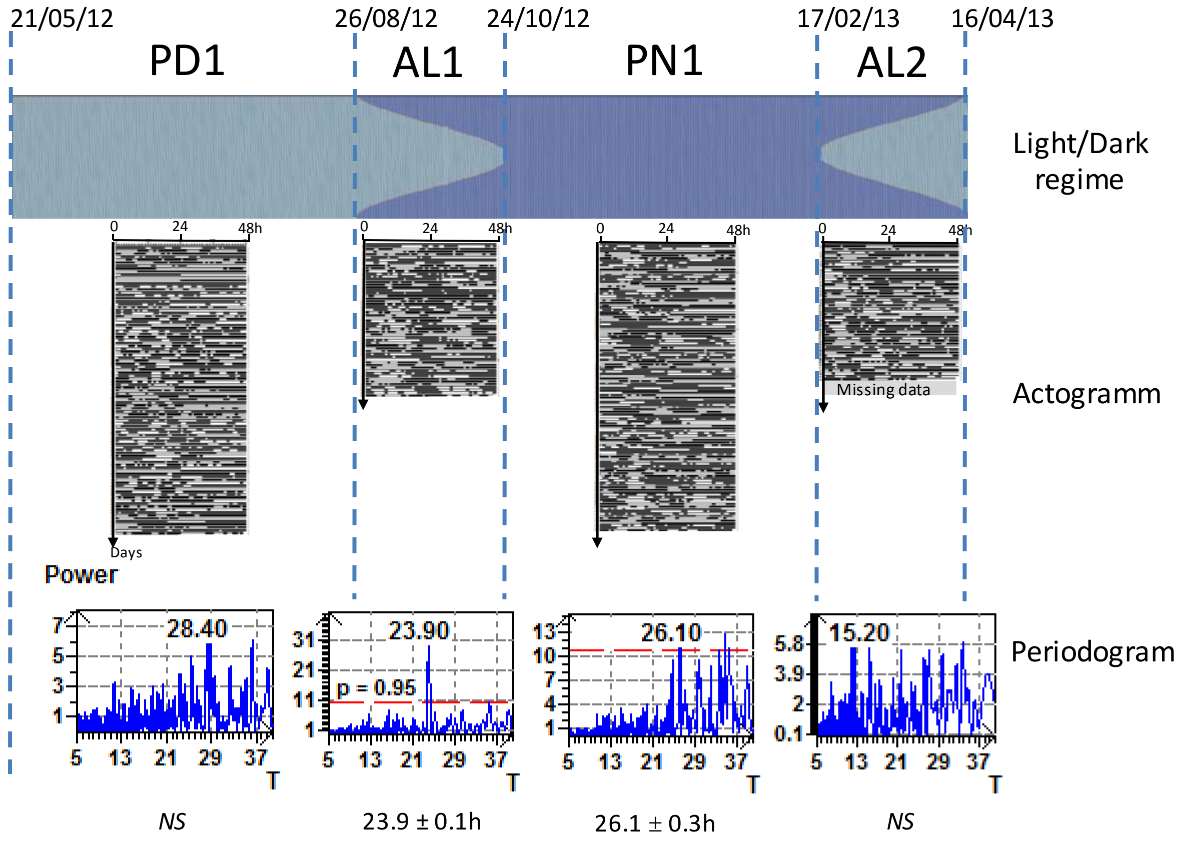
21/05/12
26/08/12
24/10/12
17/02/13
16/04/13
PD1
AL1
PN1
AL2
Light/Dark regime
0
24
48h
Days
Missing data
Actogramm
Power
28.40
23.90
p = 0.95
26.10
15.20
Periodogram
5
13
21
29
37
T
NS
23.9 ± 0.1h
26.1 ± 0.3h
NS

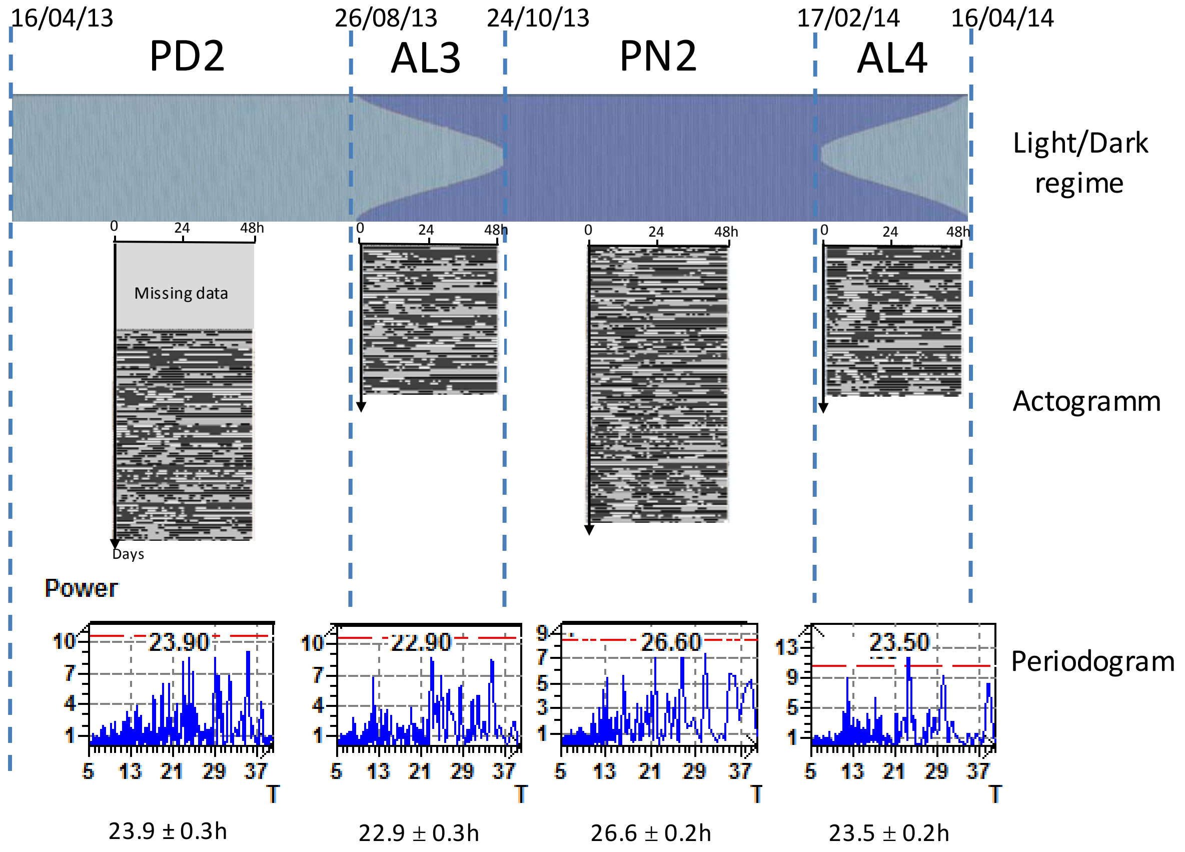

16/04/13
26/08/13
24/10/13
17/02/14
16/04/14
PD2
AL3
PN2
AL4
Light/Dark regime
Missing data
Days
Actogramm
Power
23.90
22.90
26.60
23.50
Periodogram
T
23.9 ± 0.3h
22.9 ± 0.3h
26.6 ± 0.2h
23.5 ± 0.2h

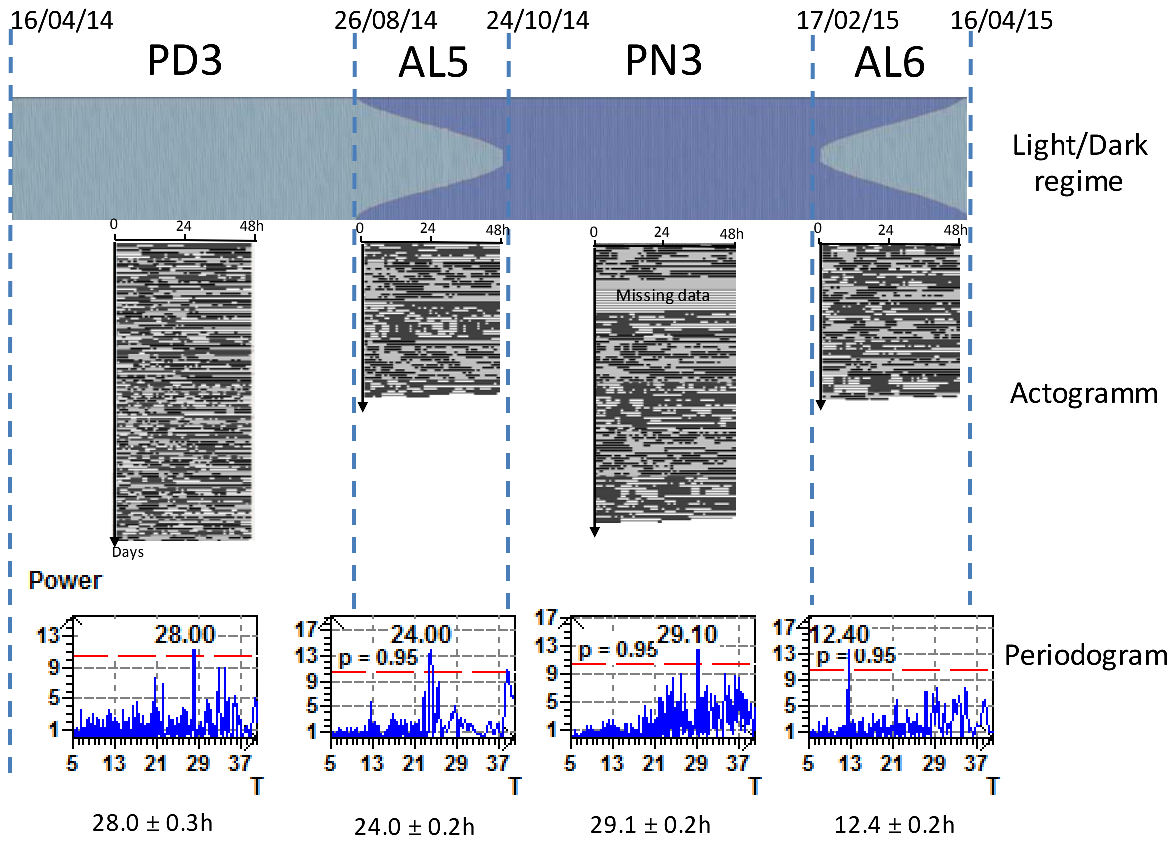

Figure SI3. *Seasonal chronobiological valve activity at mean level.*

Chronobiological analysis of the scallop *C. islandica* behavior recorded in Ny-Alesund between 21/05/2012 and 16/04/2015. In function of the light/dark regime is presented the mean behavior double-plotted actogram (each line represents 2 days) of the 14 scallops. Activity levels above the average of the day are represented by a black section, while values below the 24-h average are represented by a white section. With each actogram is shown the correspondent spectral analysis (Lomb and Scargle periodogram). If there is a significant period, its value is done below. NS: means non-significant period in the studied season.

A

Polar night periods	Civil twilight 1	Nautical twilight 1	Astrono-mical twilight	Nautical twilight 2	Civil twilight 2
Abbreviation	CV 1	NA1	AS	NA2	CV 2
Dates Ny-Alesund	24/10 09/11 *17 days*	10/11 11/12 *32 days*	12/12 30/12 *19 days*	31/12 31/01 *32 days*	01/02 17/02 *17 days*
Missing data	_	13 days 13/11/14-25/11/14	_	_	_

B

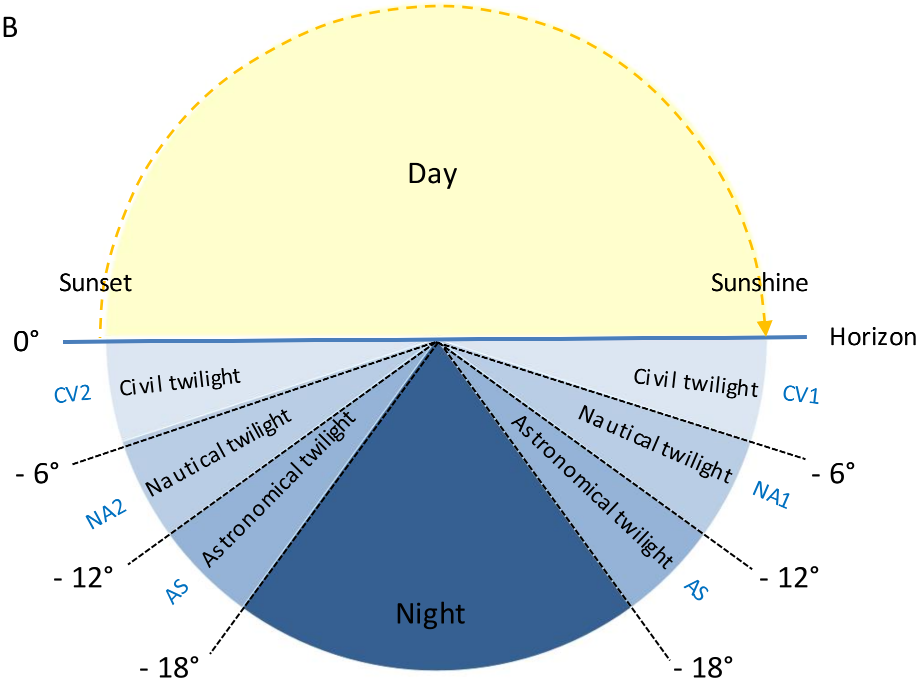

Figure SI4. *Polar night timetable and twilight periods.*

A. Timetable of twilight periods during the 3 polar nights studied. B. Scheme of twilight periods during the polar night according to the sun angle below the horizon.

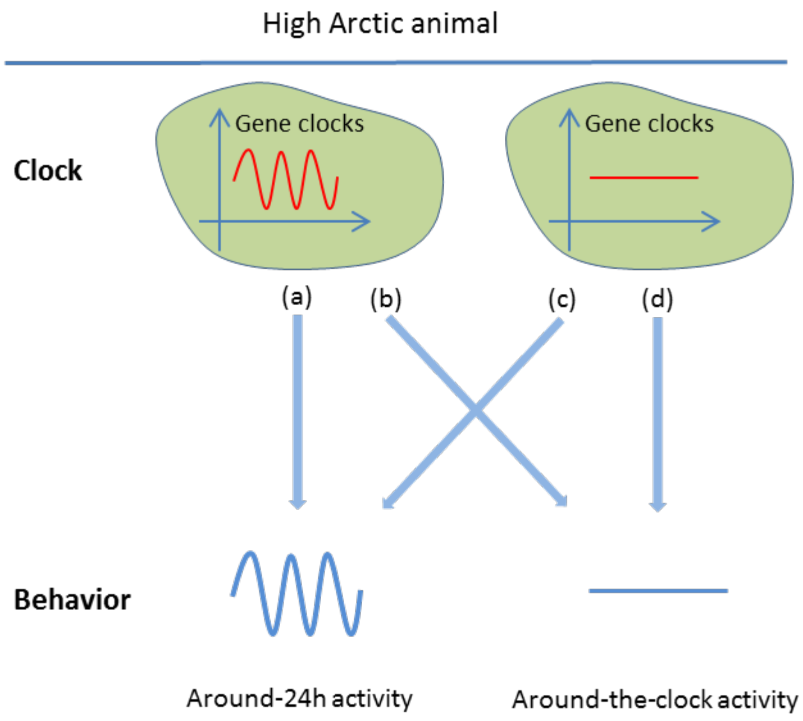

Figure SI5. *Putative mechanism underlying behavior in high Arctic regions during Polar Night*.

(a) Around 24h activity persists in PN due to the functioning circadian clock, allowing anticipating environmental change. (b) Around-the-clock activity during PN due to the decoupling of the circadian clock with physiological processes. Or the clock oscillations in PN are too low to entrain an overt rhythm. (c) Around-the-clock activity due to non-active circadian clock during PN. (d) Around 24h activity, uncoupled to non-functioning clock in PN, due to a direct response to light intensity cycles perception during the polar twilights.

Different putative strategies of animal activity in the Arctic sea.

Figure SI5 presents a synthetic diagram to illustrate four alternative scenarios (a, b, c, d). In Figure SI5 (*a*) an approximately 24 h activity persists in the PN because of a functioning circadian clock, which allows the animal to anticipate cyclic environmental changes, such as food availability. In benthic bivalves, food supply from unicellular photosynthetic organisms might convert to an autotrophic food source during the polar night[S1]. Maintaining a "true" circadian rhythm involves either a robust clock functioning without zeitgeber entrainment, such as in rodents or krills[S2-S4], or an increase in the ability of scallops to perceive light, which allows for synchronization around a 24 h cycle. Because food availability from unicellular algae is extremely low in the winter, we can infer that food availability is not the main reason to maintain a rhythm in constant light conditions. Therefore, the circadian rhythm is likely highly beneficial for the fitness of the animal and must be maintained. For example, we suspect that the maintenance of the rhythm is necessary for internal synchronization of metabolic and physiological processes[S5-S6]. Furthermore, we can also assume that the chronobiological traits observed in the Arctic scallop are under a slower process of extinction compared with other phenotypic traits[S7]. Figure SI5 (*b*) shows the around-the-clock activity during the PN caused by the decoupling of the circadian clock with behavioural activity. The loss of specific circadian behavioural activity does not necessarily mean that the circadian clock is no longer active, and the circadian activity during the PN could be an adaptation for coordinating internal metabolic processes[S7]. For example, it has been shown that in blind mammals living in caves (*Heterocephalus glaber*), the circadian clock genes oscillate with a period of 24 h without a circadian rhythm of locomotor activity[S8-S9]. An alternative explanation may be that the weak circadian pacemaker generating low clock oscillations during the PN does not allow the entrainment of overt rhythms[S10]. A third alternative explanation is proposed in Figure SI5 (*c*),

which illustrates an around-the-clock activity caused by a non-active circadian clock during the PN. The loss of circadian activity could be related to an adaptive advantage of organisms living in both varied and constant light climates throughout the year. This plasticity of the circadian rhythm has been shown in two populations of Artic reindeer. The northern reindeer population of *Rangifer tarandus platyrhynchus*, which lives at 78°N (located near Ny-Alesund, Svalbard, where the scallops' activity was recorded), shows a distinct circadian activity for locomotion during spring and autumn but an attenuated circadian activity for locomotion during the polar night and polar day. Conversely, the southern reindeer population of *Rangifer tarandus tarandus*, which lives at 70° N in northern Norway, displays a stronger circadian activity throughout the year except during the polar day[S11-S12]. Moreover, at 78° N, reindeer exhibit a non-active circadian clock with a direct activity response to light variations during the equinoxes[S13]. Similarly, a study on the bird species *Lagopus mutus hyperboreus* living in Svalbard at 79°N showed an attenuated daily oscillation in the production of the hormone melatonin (involved in the awake/sleep circadian cycle) during the polar night and polar day[S14]. This observation of the melatonin levels is consistent with a loss of a 24 h feeding rhythm during that time period[S15]. In these cases, the gradual disappearance of circadian activity corresponds to a phenotypic adaptation of the circadian clock. Furthermore, the circadian clock could be maintained for the synchronization of metabolism and become uncoupled or partially uncoupled for the locomotor behavior[S7]. Finally, the alternative in Figure SI5 (*d*) shows an approximately 24 h circadian activity that is derived from the circadian clock, which may not function during the PN. The apparent circadian rhythm is related to a direct response to the low light intensity cycles during the polar twilights. The apparent rhythm follows the decrease in light irradiance when the polar night becomes increasingly dark.

References

S1 Berge, J. *et al.* In the dark: A review of ecosystem processes during polar night. *Prog. Oceanogr.* 139, 258-271 (2015).

S2 Comas, M. & Hut, R.A. Twilight and Photoperiod Affect Behavioral Entrainment in the House Mouse (*Mus musculus*). *J. Biol. Rhythms.* 24, 403-412 (2009).

S3 Swade, R.H. & Pittendrigh, C.S. Circadian locomotor rhythms of rodents in the Arctic. *Am. Nat.* 101, 431–466 (1967).

S4 Teschke, M., Wendt, S., Kawaguchi, S., Kramer, A. & Meyer, B. A circadian clock in Antarctic krill: an endogenous timing system governs metabolic output rhythms in the euphausiid species *Euphausia superba*. *PLoS One*, 6, e26090 doi: 10.1371/journal.pone.0026090 (2011).

S5 Zhang, E.E. &, Kay, S.A. Clocks not winding down: Unravelling circadian networks. *Nat. Rev. Mol. Cell. Biol.* 11, 764–776 (2010).

S6 Green, C.B., Takahashi, J.S. & Bass, J. The meter of metabolism. *Cell*, 134, 728–742 (2008).

S7 Yerushalmi, S. & Green, R.M. Evidence for the adaptive significance of circadian rhythms. *Ecol. Lett.* 12, 970-981 (2009).

S8 Avivi, A. *et al.* Circadian genes in a blind subterranean mammal II: conservation and uniqueness of the three period homologs in the blind subterranean mole rat, *Spalax ehrenbergi* superspecies. *Proc. Natl Acad. Sci. USA.* 99, 11718–11723 (2002).

S9 Riccio, A.P. & Goldman, B.D. Circadian rhythms of locomotor activity in naked mole-rats (*Heterocephalus glaber*). *Physiol. Behav.* 71, 1–13 (2000).

S10 Bloch, G., Barnes, B.M., Gerkema, M.P., Helm, B. Animal activity around the clock with no overt circadian rhythms: patterns, mechanisms and adaptive value. *Proc. R. Soc. B.* 280, 20130019, DOI: 10.1098/rspb.2013.0019 (2013).

S11 van Oort, B.E.H. *et al.* Circadian organization in reindeer. *Nature*. 438, 1095–1096. (2005).

S12 van Oort, B.E.H., Tyler, N.J.C., Gerkema, M.P., Folkow, L. & Stokkan, K.-A. Where clocks are redundant: weak circadian mechanisms in reindeer living under polar photic conditions. *Naturwissenschaften*. 94, 183–194 (2007).

S13 Lu, W., Meng, Q.J., Tyler, N., Stokkan, K.A. & Loudon, A. A circadian clock is not required in an Arctic mammal. *Curr. Biol.* 20, 533–537 (2010).

S14 Reierth, E., Vant Hof, T.J. & Stokkan, K.-A. Seasonal and daily variation in plasma melatonin in the high-arctic Svalbard Ptarmigan (*Lagopus Mutus Hyperboreus*). *J. Biol. Rhythms*. 14, 314–319 (1999).

S15 Stokkan, K.A., Mortensen, A. & Blix, A.S. Food intake, feeding rhythm, and body mass regulation in Svalbard rock ptarmigan. *Am. J. Physiol.* 251, 264–267 (1986).
